# 

**Published:** 1896-05

**Authors:** 


### CONTENTS FOR MAY, 1896.

ORIGINAL COMMUNICATIONS. PAGE.

Amebic Catarrh of the Intestinal Tract. By A. A. YOUNG, M. D. .... 769
Establishment and Early Days of the Medical Department of the University of Buffalo. By C. C. WYCKOFF, M. D. .... 773
Hysterectomy in Puerperal Sepsis. By HERMAN E. HAYD, M. D. .... 777
Establishment of the Medical Department of Niagara University. By JOHN CRONYN, M. D. .... 780
Report of a Case of Otitic Brain Abscess, with Remarks on Diagnosis. By ALVIN A. HUBBELL, M. D. .... 782
The Symptomatology and Pathology of Exophthalmic Goitre. By WM. C. KRAUSS, M. D. 793

CLINICAL REPORT.

Placenta Previa .... 801

PROGRESS IN MEDICAL SCIENCE.

Ophthalmology and Otology .... 804
Nervous Diseases and Insanity .... 809
Public Health, Sanitation and Vital Statistics .... 812
Obstetrics, Gynecology and Pelvic Surgery .... 814

NEW INSTRUMENTS.

Cervical Tractor and Guide, for Trachelorrhaphy .... 820

EDITORIAL.

A Woman's Edition .... 822
Medical Department of Niagara University .... 823
Topics of the Month .... 824

---

Personal .... 826
Obituary .... 827
Society Meetings .... 828
Medical College and Hospital Notes .... 834
Reviews .... 835
Literary Notes .... 847

